# Kyste hydatique pelvien primitif: à propos d’un cas

**DOI:** 10.11604/pamj.2016.25.239.11238

**Published:** 2016-12-20

**Authors:** Jamal Bouihi, Houda Moustaide, Bouchra El Amrani, Ahmed Mimouni

**Affiliations:** 1 Service de Gynécologie-Obstétrique, CHU Mohammed VI, Oujda, Maroc

**Keywords:** Hydatidose, parasitose, kyste, pelvis, chirurgie, Hydatidosis, parasitosis, cyst, pelvis, surgery

## Abstract

L’hydatidose sévit à l’état endémique au Maroc, sa localisation pelvienne est rare et trompeuse. Nous rapportons l’observation d’une patiente âgée de 24 ans admise pour une énorme masse pelvienne associée à une pesanteur sans autres signes accompagnateurs. L’échographie et le scanner ont permis de localiser une masse anéchogène cloisonnée latéro- et sus-utérine droite. Le diagnostique de kyste hydatique pelvien est fait en peropératoire. Le traitement a consisté en une résection du dôme saillant. L’évolution était favorable avec un recul de trois ans.

## Abstract (en)

Hydatidosis is endemic in Morocco, its pelvic localization is rare and deceptive. We report the case of a 24-year old patient admitted with a giant pelvic mass associated with heaviness with no further accompanying signs. Ultrasound and CT scan allowed localization of a right lateral and subuterine septated anechoic mass. The diagnosis of pelvic hydatid cyst was made intraoperatively. Treatment consisted of resection of the protruding dome. Patient’s evolution was favorable, with a follow-up period of three years.

## Introduction

L’hydatidose est une maladie due au développement dans l’organisme humain de la forme larvaire d’un taenia du chien: *Echinococcusgranulosus* [1, 2]. Le Maroc, pays d’élevage traditionnel, se place parmi les pays les plus infestés par cette parasitose [1, 2]. Les localisations hépatiques et pulmonaires sont les plus fréquentes (1,3). Les kystes hydatiques à localisation pelvigénitale chez la femme font partie de ces cas rares et trompeurs [3, 4]. Le diagnostic basé sur les examens radiologiques etsérologiques est parfois difficile [1, 3, 4]. Nous rapportons une observation de kyste hydatique pelvien qui nous a paru intéressante à documenter en raison d’un tableau clinique simulant tumeur ovarienne.

## Patient et observation

Il s’agit d’une patiente âgée de 24 ans, célibataire, sans antécédent pathologique particulier, le début de ses symptômes remonte à quatre mois avant son admission par une douleur pelvienne à type de pesanteur d’aggravationprogressive associé à une augmentation progressivedu volume abdominopelvien, sans troubles urinaires ou digestifs associés, évoluant dans un contexte d’apyrexie et conservation de l’état général.

**Figure 1:**
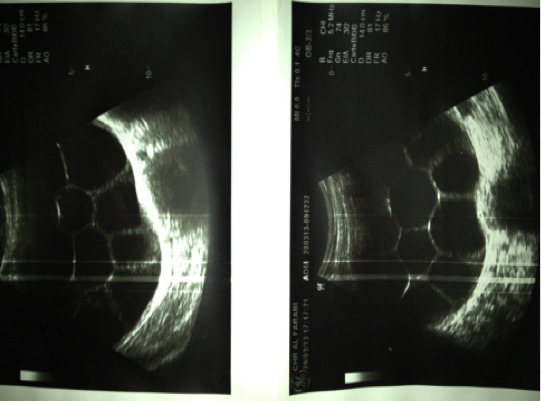
Enorme masse anéchogène, occupant la totalité du pelvis avec des cloisonsintrakystiques en nid d’abeilles, sans végétations intra ou exokystiques

**Figure 2:**
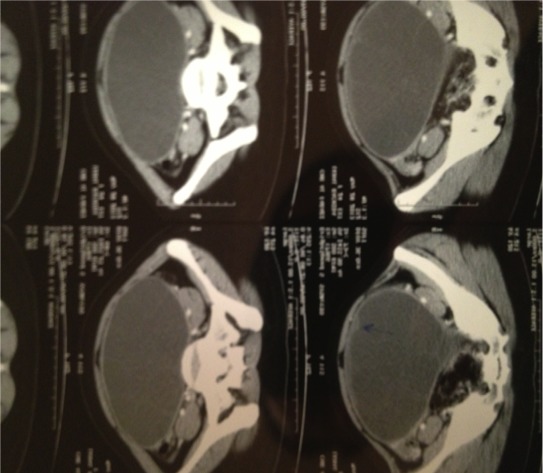
TDM: masse pelvienne supra et latéro- utérine, liquidienne, mulicloisonées avec réh- des cloisons et des végétations endokystiques mesurant jusqu’au 20 mm d’épaisseur

**Figure 3:**
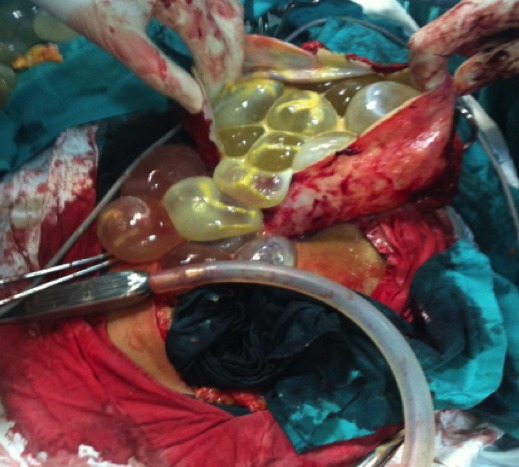
Rupture accidetelle de la masse lors de la liberation de ses adherences

**Figure 4:**
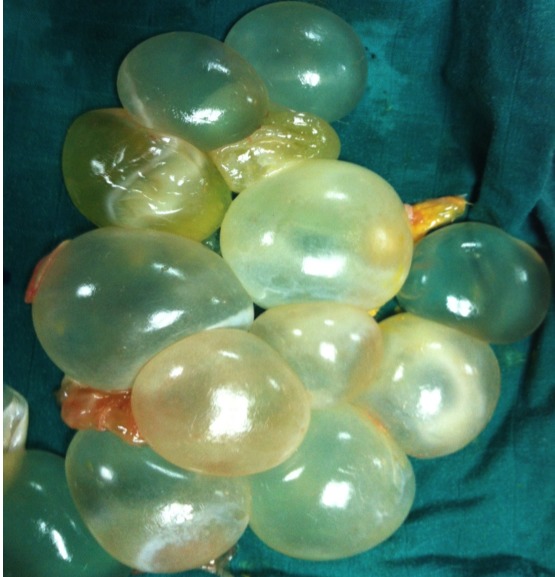
Les vesicules filles issues de la masse

L’examen clinique à trouvé une masse abdominopelvienne médiane, mesurant environ 30 cm de diamètre, arrivant à plus de trois travers de doigts au dessus de l’ombilic, légèrement mobilisable, de consistance ferme, bien limitée, sensible. L’échographique a mis en évidence une énorme masse anéchogène, occupant la totalité du pelvis avec des cloisons intrakystiques en nid d’abeilles, sans végétations intra ou exokystiques ou épanchement péritonéal (Figure 1). La tomodensitométrie pelvienne a montré une *masse pelvienne supra et latéro-utérinedroite, liquidienne, multicloisonées avec réhaussement des cloisons et des vegétations endokystiques mesurant jusqu’au 20 mm d’épaisseur* (Figure 2). La radiographie pulmonaire et l’échographie hépatique étaient sans anomalie. Le taux du CA125 était normal. L’exploration chirurgicale a trouvé un kyste pelvien de 30 cm de diamètre adhérent aux organes de voisinage, L’exploration péritonéale à la recherche d’une autre localisation était négative. Une rupture accidentelle s’est produite, avec issue de multiples vésicules filles Lors de la libération de la masse de ses adhérences, (Figure 3, Figure 4). L’intervention chirurgicale a été réalisée avec toutes les mesures de protection pour éviter une éventuelle dissémination secondaire. Une résection du dôme saillant a été faite. L’étude anato-mopathologique était non concluante. Les suites postopératoires étaient simples. Devant ce tableau ambiguë, Une sérologie hydatique à été demandée, elle s’est révélée positive, La patiente a été mise sous traitement médicale a base d’albendazole, actuellement, elle est suivie en consultation de gastroentérologie, avec un recul de trois ans sans récidive locale oupéritonéale.

## Discussion

La maladie hydatique est fréquente au Maghreb, où elle sévit à l’état endémique. Elle se localise en n’importe quel point de l’organisme, dès que les filtres hépatique et pulmonaire sont dépassés [1, 3, 5]. L’hydatidose pelvienne est rare. Son incidence est comprise entre 0,30 et 4,27% des localisations hydatiques selon les auteurs [1, 6, 7], dont 80% des cas implique la sphère génitale [3]. La majorité des cas rapportés dans la littérature concerne des patientes âgées entre 20 et 40 ans [1, 3].

Le mode de contamination hydatique de la région pelvienne demeure hypothétique; la contamination est habituellement secondaire à la rupture intra-abdominale d’un kyste hydatique hépatique; les vésicules filles et les scolex libérés se fixent dans le cul de sac de douglas et continuent leur développement; une endothélialisation secondaire les exclut de la cavité péritonéale; ainsi, le kyste intrapéritonéal devient extrapéritonéal et semble faire partie du tissu cellulaire pelvien [1, 2, 3, 6]. Cependant, des kystes hydatiques pelviens primitifs ont été rapportés, comme c’est le cas chez notre patient [3, 8]. Elle est due à une contamination par voie hématogène, suggérant une rup- ture du filtre hépatique ou pulmonaire par le parasite pour avoir accès à la grande circulation. Cette forme ne peut être retenue que si la patiente ne présente aucune autre localisation hépatique, pulmonaire, splénique… [1, 3, 8].

Par ailleurs, le diagnostic de l’hydatidose pelvienne est exceptionnellement porté en pré opératoire puisque l’hydatidose pelvienne est une affection rare et trompeuse. L’échinococcose pelvienne est le plus souvent révélée par une masse abdominopelvienne associée à des douleurs pel- viennes [1, 4, 9].

Elle peut également être révélée par des signes de compression des organes de voisinage [1–4] ou une complication aiguë telle une rétention d’urine ou une anurie par compression bilatérale des uretères [1]. La symp- tomatologie peut être vague et déroutante, telle que des métrorragies ou une stérilité. Le diagnostic peut être posé fortuitement lors de la césarienne ou lors d’une grossesse compliquée, un travail dystocique ou une hémorragie des suites de couche [10]. La symptomatologie est très poly- morphe, il n’y a pas de signe spécifique ou évocateur, d’où l’intérêt qu’il faut donner à l’interrogatoire et aux examens paracliniques[1, 3]. La maladie demeure habituellement asymptomatique pendant des années et n’est découverte de fa ?con fortuite que lors d’un examen clinique ou radiologique [1–3].

L’échographie est primordiale devant toute masse abdominopelvienne. Elle permet la précision des caractères de la masse, ses relations avec les organes de voisinage et d’explorer le reste de l’abdomen à la recherche d’une autre localisation notamment hépatique.

Les images échographiques du kyste hydatique pelvien sont identiques à celles décrites au niveau du foie et classées en cinq types [1, 2, 5, 7]: type I: collection liquidienne pure bien limitée; type II: collection liquidienne à paroi dédoublée; type III: collection liquidienne multivésiculaire; type IV: masse d’échostructure hétérogène à prédomi- ?nance liquide ou solide; type V: kyste calcifié.

L’image kystique de notre patiente est classée type III. La tomodensitométrie trouve son indication devant les limites de l’échographie [1]. La radiographie du thorax permet la recherche d’une éventuelle localisation pulmonaire. L’imagerie par résonance magnétique permet l’analyse des rapports pelviens du kyste hydatique inaccessible à la tomodensitométrie. Elle permet aussi en cas de doute de faire le diagnostic différentiel avec les tumeurs périrectales, d’une part, et vestigiales, d’autre part, nerveuse ou osseuse [1]. L’urographie intraveineuse permet surtout la recherche d’un retentissement sur le haut appareil. Il n’y a aucun examen sérologique ou immunologique pathognomonique de la maladie hydatique [3]. La place accordée à l’immunologie est un atout capital surtout pour la surveillance postopératoire. Ce sont des tests d’hémagglutination et d’immunofluorescence qui sont les plus sensibles. L’immunoélectrophorèse est plus spécifique. Toutefois, ces tests sont négatifs dans 10 % des cas surtout pour les kystes jeunes et univésiculaires et le diagnostic reste souvent une découverte peropératoire [1, 3, 4, 5, 7]. Le diagnostic différentiel se fait avec toutes les tumeurs kystiques ou mixtes rétro péritonéales (kystes dermoïdes), les abcès à pyogènes ou les abcès tuberculeux, les kystes de l’ovaire ou les hydrosalpinx, les tumeurs ovariennes ou les fibromes utérins surtout sous séreux [1, 3, 7, 8, 10]. La chirurgie est le traitement de choix de l’hydatidose pelvienne. La kystectomie totale est le procédé idéal, mais la kystectomiepartielle ou subtotale peut être réalisée pour éviter de blesser les organes de voisinage [1, 3, 5–7]. Les agents scolocides les plus utilisés sont le NaCl ou l’eau oxy- génée. L’exploration doit rechercher d’autres localisations hydatiques qui seront traitées en même temps. Le mében- dazole ou l’albendazole sont utilisés entraitement adjuvant de la chirurgie pour minimiser les récidives.

Un recul de deux ans est nécessaire pour juger de l’efficacité du traitement [1, 3, 6, 7, 10]. L’idéal dans un pays d’endémie comme le nôtre où l’hydatidose constitue un problème de santé publique est de développer les moyens de prophylaxie dont le substratum essentiel est la lutte contre l’infestation de l’hôte définitif, la protection de l’hôte intermédiaire et la lutte contre la contamination de l’Homme.

## Conclusion

La localisation primitive du kyste hydatique au niveau du pelvis est exceptionnelle chez la femme, sa symptomatologie clinique déroutante. Le diagnostic préopératoire de l’hydatidose pelvienne est parfois difficile. Le diagnostic doit être évoqué devant tout processus occupant l’espace pelvien, surtout dans un pays d’endémie. La chirurgie est le traitement de choix. Mais la prévention est le meilleur moyen pour diminuer l’incidence de cette pathologie. Points essentiels: l’hydatidose est une maladie fréquente en zone d’endémie; la localisation pelvienne est rare; le diagnostic préopératoire est difficile; elle doit être évoquée devant toute masse pelvienne chez une femme originaire d’une zone d’endémie; le traitement est essentiellement chirurgical.
